# High Temperature Increases the Masculinization Rate of the All-Female (XX) Rainbow Trout “Mal” Population

**DOI:** 10.1371/journal.pone.0113355

**Published:** 2014-12-12

**Authors:** Karina Valdivia, Elodie Jouanno, Jean-Nicolas Volff, Delphine Galiana-Arnoux, René Guyomard, Louise Helary, Brigitte Mourot, Alexis Fostier, Edwige Quillet, Yann Guiguen

**Affiliations:** 1 INRA, UR1037 LPGP Fish Physiology and Genomics, F-35000, Rennes, France; 2 IGFL, UMR5242 CNRS/INRA/Université Claude Bernard Lyon I/ENS, Lyon, Cedex 07, France; 3 INRA, UMR1313 GABI Génétique Animale et Biologie Intégrative, Domaine de Vilvert, 78352, Jouy-en-Josas Cedex, France; Temasek Life Sciences Laboratory, Singapore

## Abstract

Salmonids are generally considered to have a robust genetic sex determination system with a simple male heterogamety (XX/XY). However, spontaneous masculinization of XX females has been found in a rainbow trout population of gynogenetic doubled haploid individuals. The analysis of this masculinization phenotype transmission supported the hypothesis of the involvement of a recessive mutation (termed *mal*). As temperature effect on sex differentiation has been reported in some salmonid species, in this study we investigated in detail the potential implication of temperature on masculinization in this XX *mal*-carrying population. Seven families issued from XX *mal*-carrying parents were exposed from the time of hatching to different rearing water temperatures ((8, 12 and 18°C), and the resulting sex-ratios were confirmed by histological analysis of both gonads. Our results demonstrate that masculinization rates are strongly increased (up to nearly two fold) at the highest temperature treatment (18°C). Interestingly, we also found clear differences between temperatures on the masculinization of the left versus the right gonads with the right gonad consistently more often masculinized than the left one at lower temperatures (8 and 12°C). However, the masculinization rate is also strongly dependent on the genetic background of the XX *mal*-carrying families. Thus, masculinization in XX *mal*-carrying rainbow trout is potentially triggered by an interaction between the temperature treatment and a complex genetic background potentially involving some part of the genetic sex differentiation regulatory cascade along with some minor sex-influencing loci. These results indicate that despite its rather strict genetic sex determinism system, rainbow trout sex differentiation can be modulated by temperature, as described in many other fish species.

## Introduction

The primary causal signal responsible for sex determination in vertebrates is variable [1]. Two main types of primary sex determination have been described in gonochoristic species: genotypic sex determination (GSD) and environmental sex determination (ESD). This sex determination switch (either GSD or ESD) will then trigger the gonadal sex differentiation process with the development of testes or ovaries from undifferentiated gonads. In GSD, the sex of the embryo is strictly determined by the genotypic information inherited from its parents (i.e., mammals and birds). In ESD, the sex of the embryo is physiologically determined by the environment (i.e., some reptiles and some fish species) [1]. Intermediate sex determination systems have also been described [2], in which a number of genetic factors and environmental influences both contribute to the determination of the final sex-ratio. This is the case for species with minor genetic factors that override the sex chromosomes, such as tilapia, *Oreochromis niloticus* [3], [4] or for species with polygenic sex determination, such as sea bass, *Dicentrarchus labrax* [5]. The best-characterized ESD to date is a temperature-dependent sex determination (TSD) that has been well described in some reptiles, amphibians [6] and fish species [7]. In teleost fish, a large range of sexuality types has been described, from hermaphroditism to gonochorism [8]. This variability is due to the high diversity of sex determination systems [9] that include several stages between full GSD and full ESD [10], [11]. A detailed review on the influence of temperature [7] on sex differentiation in teleosts distinguished a full and physiological TSD system from the thermal effects on GSD. Remarkably, in both cases, high temperatures consistently induced male-biased sex ratios [7]; with only a few notable exceptions, including the sockeye salmon, *Oncorhynchus nerka*, in which a female-biased sex ratio was observed at high temperatures [12]. However, these results were not confirmed by a follow-up study on other strains of the same species [13]. Although studies on salmonids indicate they have a simple and robust XX/XY GSD system [14], [15], thermal effects on GSD have also been observed in at least two *Oncorhynchus* species, the sockeye salmon as mentioned above [12], [13] and the rainbow trout, *O. mykiss* [16]. Initial studies on rainbow trout did not detect any effect of high temperatures on sex differentiation either with short-term [17] or long-term treatments [18]. However, a more recent and thorough study on rainbow trout demonstrated that high temperatures (18°C) could modulate sex ratios with slight but significant deviations from a balanced sex-ratio [16]. Interestingly, these minor sex-ratio deviations were detected either in favor of males or of females according to the genetic background of the tested population. These results have since been confirmed and the frequency of masculinization in response to temperature was shown to be a heritable trait [19]. Despite a strict male heterogamety in rainbow trout, spontaneous masculinization has been found in some XX fish belonging to a population of gynogenetic doubled haploid individuals [20]. Analysis of the transmission of this male phenotype in a three generation pedigree supported the hypothesis that a recessive mutation in one putative minor sex determination factor (termed *mal*), together with other sex modifier loci, was responsible for the partial or full masculinization of some of these XX individuals [20]–[22]. Preliminary observations also suggested that this expression of maleness was influenced by environmental factors; low temperature during the first stages of development reduced the frequency of masculinized individuals [Quillet et al., unpublished data]. In this study, we investigate in greater detail the potential role of temperature on masculinization in *mal*-carrying rainbow trout individuals. Seven families issued from *mal*-carrying parents were exposed during the sex differentiation period to different rearing water temperature (8, 12 and 18°C). The resulting sex-ratios derived from the various temperature treatments were assessed by gonadal histology and compared. Our results confirm that the masculinization rate of rainbow trout depends on the genetic background. Furthermore, we demonstrated that masculinization of XX *mal*-carrying individuals is strongly increased during a high temperature treatment (18°C). These results suggest that despite its rather strict GSD system, thermal effects in combination with special genetic backgrounds can influence rainbow trout sex differentiation, a developmental trait described in many other fish species.

## Materials and Methods

### Ethics Statement

Research involving animal experimentation has been approved by the Fish Physiology and Genomics Laboratory (INRA LPGP, Rennes) experimental facilities' ethical committee (authorization number 12I08) and the author's institution (authorization number 35–14). All experiments performed on animals in this study complied with French and European regulations regarding the use and care of laboratory animals. All analyses were performed to minimize suffering. Fish were always sampled under 2-phenoxyethanol anesthesia (0.3 ml/l of water) and were euthanized by a lethal dose of anesthesia (1 ml/l of water).

### Animals

The experimental XX *mal*- carrying line studied here was derived from the winter-spawning INRA-SY (Synthetic) rainbow trout strain that is maintained at the INRA experimental fish facilities (PEIMA, France). It was originally established from a single *mal*- carrying female (namely the D12 female as described in [20]). Briefly, gynogenetic reproduction of the D12 female produced XX doubled haploid males (generation G1). In order to limit inbreeding, those XX males were mated with standard SY females to produce the next generation (G2). The G3 generation was obtained by mating G2 females with G2 spontaneous males (plus one doubled haploid progeny directly derived from D12). The *mal*- carrying line was further maintained by subsequent within-line crosses between females and spontaneous phenotypic males. Two sets of experimental progeny were analyzed in this study. In experiment A (November 2008), four experimental half-sib families of *mal*-carrying XX rainbow trout (mal1 to mal4) were generated by mating four G3 females with one doubled haploid male from D12. In experiment B (November 2010), three additional full-sib families of *mal*-carrying XX rainbow trout (mal5 to mal7) were generated by single pair mating of three G4 females and three G4 males. An all-female (XX) group was also produced as a control in experiment B using standard XX neomales as previously described [23], [24]. Males and females used to produce those controls were issued from the Autumn INRA line.

Fish were fed with a commercial feed. The feeding level was calculated as a percentage of the total tank biomass. This percentage was adjusted to the growth of the animals that were weight every two weeks. This daily ration corresponded to 3.8% of the biomass for a mean individual weight below 0.6 g, 3.5% between 0.6 and 1.5 g, 3.1% between 1.5 and 5 g, 2.6% between 5 and 15 g, 2.0% between 15 and 50 g, 1.7% between 50 and 200 g, 1.2% between 200 and 500g and 1.0% between 500 g and 1 kg.

### Temperature treatments

**Figure 1:**
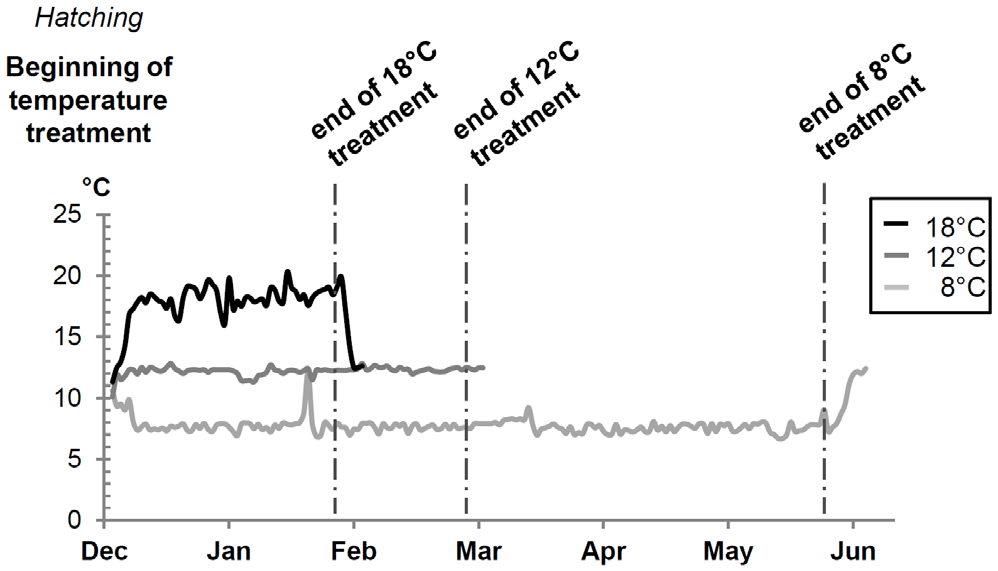
Experimental conditions for the common garden experiment A. Eggs from four *mal*-carrying progeny were incubated at 10°C until hatching and were then shifted to the 8, 12 or 18°C temperature treatment. At first feeding, the total number of fish per progeny was reduced to 110 and all four progeny were mixed for each temperature condition. At the end of the temperature treatment, the fish were transferred and maintained at 12°C (see the Materials and Methods section for more details).

In the common garden experiment A (families mal1 to mal4), batches of 600 eggs per family were incubated at 10°C±1°C until hatching (approximately 32 days post-fertilization) and then transferred to indoor experimental facilities at the Fish Physiology and Genomics Laboratory (INRA LPGP, Rennes). Each batch was split into three groups of 200 hatched embryos that were maintained separately in incubators at three different temperatures (Fig. 1): 8, 12 and 18°C (calculated mean values: 7.8±0.8°C; 12.1±0.4°C; 17.9±1.7°C, see also S1 Figure). At 560 days post-fertilization, *i.e.* at the free swimming and first feeding stage, the fish were transferred to tanks and 110 progeny fish from each family were pooled into three separate tanks resulting in three distinct progeny common garden experiments (one per temperature). To partially compensate for growth differences, the duration of each thermal treatment was adjusted according to the temperature: 6 months at 8°C (1440 degree-days), 3 months at 12°C (1080 degree-days) and 2 months at 18°C (1080 degree-days). At the end of each thermal treatment, the fish were maintained at 12°C until they attained a sufficient size for sexing (between 9 and 12 months post-hatching depending on the initial temperature regime) [25], [26]. Fish were sacrificed with a lethal dosage of phenoxyethanol (0.1% in water). For each individual, both left and right gonads were fixed in Bouin-Holland fluid for histological analysis and a fin clip was stored in 90% ethanol for subsequent genotyping and parental assignment. The original breeders were also fin clipped. In experiment B, the all-female control group and the three XX *mal-*carrying progeny were cultured in separate tanks and exposed to 2 temperature treatments (12°C and 18°C) for 2 months post-hatching, then maintained at 12°C until they grew sufficiently for sexing. Between 50 and 60 fish per experimental group were sacrificed at 7 months old, and their gonads were sampled for histological analysis.

### Histological analysis

Gonads were fixed for 48 hr in Bouin-Holland fluid and then dehydrated serially in aqueous 70% and 95% ethanol, ethanol/butanol (5∶95) and butanol. Tissues were embedded in paraffin, and 5-µm longitudinal gonad sections were stained with Regaud's hematoxylin [27].

### Sex-ratio assessment

Fish gonadal phenotypes were analyzed and classified as described in Valdivia et al. [22] (see S2 Figure). Briefly, the fish were scored as “normal female” when no sign of masculinization could be detected by histological analysis on either gonad and when no delay in gametogenesis could be observed compared to the control females (XX all-female population). Females with delayed ovarian development were scored as “delayed oogenesis females”. All other phenotypes exhibiting masculinization features were classified as either “normal males” or “intersex animals.” The animals that received intersex scores had at least one gonad with some signs of masculinization. The sex-ratio in experiment A was assessed only for fish correctly assigned to a progeny an in which the histology was available on both gonads.

### Genotyping and parental assignation

For the common garden experiment (experiment A), the pedigree of each progeny from the 4 experimental *mal*-carrying families was recovered by the genotyping of 13 microsatellite markers (OMM1013, OMM1050, OMM1117, OMM1313, OMM1354, OMM1449, OMM5013, OMM5043, OMM5098, OMM5126, Omy7INRA, Omy77 and Ots1BML). DNA extraction from the parents and progeny fin clips and genotyping was carried out at LABOGENA (http://www.labogena.fr/, Jouy-en-Josas, France). Parental assignment was then performed by exclusion using the VITASSIGN software [28]. At the end of the process, 97% of the genotyped offspring were unambiguously assigned to their maternal family. The presence of some unassigned individuals affected the accuracy of survival estimates within the different families.

### Statistical analyses

Fisher's exact test was used to compare the mortality rates between temperature treatments. The influence of temperature on the occurrence of the different sexual phenotypes was assessed using a generalized linear mixed model assuming that the frequency of a given phenotype depends on effects of temperature and family modeled as fixed and random effects respectively. Analyses were performed with SAS Glimmix procedure for each phenotype (gonadal phenotype modeled as a binary trait, logit scale as the link function). Thus, the frequency of a given phenotype in family *i* at temperature A, *P_iA_*, is related to temperature and family effects as follows: log[*P_iA_*/(1- *P_iA_*)]  = β_0_+β_A_+γ*_i_* ; where β_0_ and β_A_ correspond to the temperature effect, and γ*_i_* is a random variable corresponding to the random selection of families (SAS Glimmix procedure, http://www.ats.ucla.edu/stat/sas/glimmix.pdf). Family differences were assessed from experiment A only (common garden experiment) as they were mixed with tank effect in experiment B.

## Results

### Overall effect of temperature on the masculinization rate in *mal*-carrying animals

**Figure 2:**
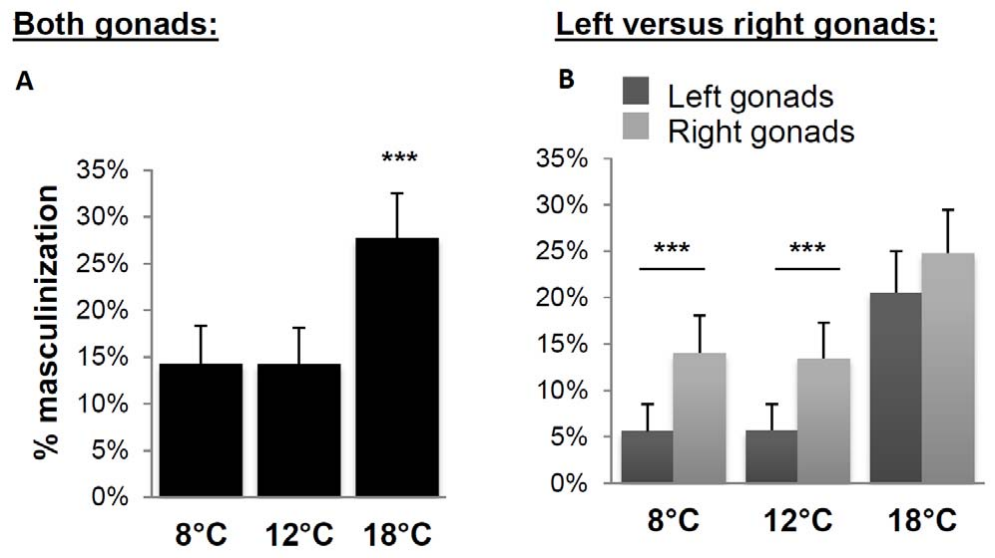
Overall effect of different rearing temperatures on the masculinization rate in Experiment A (pool of four different *mal*-carrying progeny). Panel A: Individual masculinization rates (each individual is considered masculinized when at least one gonad is masculinized) different temperature treatments (8, 12 and 18°C) (in percentage ± Confidence Interval at p = 0.05; χ^2^; *** p<0.001). Panel B: Masculinization rates of left versus right gonads following different temperature treatments (8, 12 and 18°C) (in percentage ± Confidence Interval at p = 0.05; χ^2^; *** p<0.001). Numbers of animals analyzed at 8°C n = 357, 12°C n = 386 and 18°C n = 375.

In experiment A, the average mortality rate was 10.6%, with a significantly higher mortality rate at 8°C than at either 12°C or 18°C (Table 1). Subsequent analyses were performed on animals that were both successfully assigned to a family and for which both gonads were analyzed by histology (94.4%, 95.8% and 94.0% of the initial number of fish sampled for the 8°C, 12°C and 18°C treatments, respectively). A marked effect of temperature on the overall sex ratios was observed (Table 2). Altogether, 73.9%, 75.1% and 63.2% of the available fish were recorded as normal previtellogenic females at 8°C, 12°C and 18°C respectively, corresponding to a significant overall effect of rearing temperature (P = 0.021). However, only high temperature (18°C) was associated with significant differences in frequency of gonadal phenotypes while no significant difference between 8°C and 12°C was recorded. This effect was first characterized by a marked reduction of the proportion of normal females at 18°C (P = 0.011) compared to 8°C and 12°C. A small proportion of females with delayed oogenesis (overall mean  = 10.5%) was also recorded in all groups irrespective of the rearing temperature (P = 0.51). The percentage of total masculinization (intersex plus male individuals) varied according to temperature treatment in the opposite direction to females (P<0.004) with a two fold increase of masculinized individuals at 18°C when compared to 8°C and 12°C (27.7% and 14.3% respectively, P = 0.003). The most frequent phenotype among the masculinized individuals was the intersex type rather than full males, regardless of temperature (Table 2). Additionally, we found that the right gonad was consistently more often masculinized than the left one. However, this difference was statistically significant only at the 8°C and 12°C treatments (Chi-2 test see Fig. 2B).

**Table 1:** Overall mean survival rates and assignment success according to temperature treatments in experiment A.

Mean survival and assignment rate	8°C	12°C	18°C
Total number of fish at the end of the experiment	378	403	399
Mortality rate (% of initial number of fish)	14.1*	8.4	9.3
Genotyping failure (%)	3.9	2.9	2.0
Final number of fish available (assigned and both gonads observed)	357	386	375

* : mortality significantly higher at 8°C (*P<0.05*).

**Table 2:** Overall mean frequency of the different gonadal phenotypes according to temperature treatments in experiment A.

Gonadal phenotype (%)	8°C	12°C	18°C	Temperature effect^1^
Previtellogenic females (%)	73.9 a	75.1 a	63.2 b	*P = 0.021*
Females with delayed oogenesis (%)	11.7	10.6	9.1	*P = 0.515*
Intersex (%)	12.6 a	13.5 a	21.1 b	*P = 0.035*
Males (%)	1.7 a	0.8 a	6.7 b	*P = 0.013*
Total masculinization rate (intersex + males, %)	14.3 a	14.3 a	27.8 b	*P = 0.004*
Total number of fish examined	357	386	375	

1 : Glimmix analysis performed with for each phenotype the complete set of data (3 temperatures, 4 families) on the logit scale assigning binary gonadal phenotypes (1 =  individual with the target phenotype, 0 =  other individuals) and temperature and family effects modeled as fixed and random effects respectively. Within line: different subscripts (a, b) indicate significant differences among temperatures for the proportion of the gonadal phenotype (paired comparisons of temperature using Glimmix analysis, *P<0.05*).

In view of the results obtained in experiment A, a second experiment (B) was designed to confirm the effects of a high rearing temperature (18°C) on masculinization rates by testing three additional *mal*-carrying families at 18°C versus 12°C (Table 3). At 12°C, the rate of masculinization was much higher than in experiment A (73.3% as an overall mean instead of 14.3%), but this rate was still significantly increased at 18°C whatever the family (97.1% as an overall mean, P<0.001, Table 3). Conversely to what was observed in experiment A, the relative frequency of full males was similar or higher than the frequency of intersex individuals. A combined analysis of data from experiment A (8°C excluded) and experiment B throughout the 7 families of the design confirmed the masculinizing effect of high temperature in XX mal-carrying progeny (12°C versus 18°C, data not shown). In contrast, we did not observe any masculinization in the all-female XX control population whatever the temperature (12°C or 18°C).

**Table 3:** Effect of temperature treatment on the masculinization rate in Experiment B.

Temperature	12°C				18°C				Temperature effect
Gonadal phenotype	intersex	male	n_T_	Masculinization rate (%)	intersex	male	n_T_	Masculinization rate (%)	
All-female control	-	-	100	0	-	-	100	0	*ns*
mal5	21	24	63	71.4	14	38	53	98.1	*P<0.001* 1
mal6	23	17	49	81.6	25	39	66	96.9	*P<0.05* 1
mal7	21	15	53	67.9	36	17	55	96.3	*P<0.001* 1
Total	65	66	165	73.3	75	94	174	97.1	*P<0.001* 2

Intersex, male: number of each phenotype in the family; n_T_: total number of fish in the family;

1 χ^2^ test (df = 1) for effect of temperature on total masculinization rate within each family;

2 results of Glimmix analysis performed with the whole set of data (2 temperatures, 3 families) on the logit scale assigning binary gonadal phenotypes (1 =  intersex or males, 0 =  females) and temperature and family modeled as fixed and random effects respectively.

### Family effect on the masculinization rate in *mal*-carrying animals

**Figure 3:**
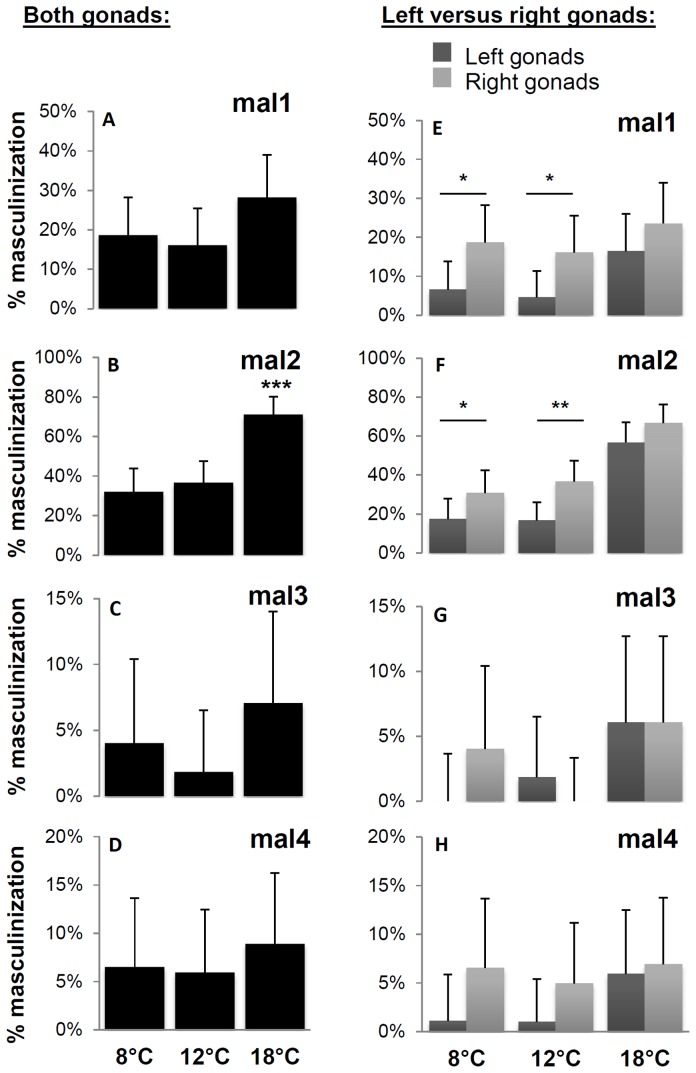
Rates of masculinization of the different *mal-carrying* progeny exposed to different temperatures in experiment A. Panels A to D: Individual masculinization rates (each individual is considered masculinized when at least one gonad is masculinized) following different temperature treatments (8, 12 and 18°C). Panels E to H: Masculinization rates of left versus right gonads following different temperature treatments (8, 12 and 18°C) (in percentage ± Confidence Interval at p = 0.05%+CI; χ^2^; * p<0.05, ** p<0.01, *** p<0.001). Numbers of animals analyzed at 8, 12 and 18°C, respectively: mal1  = 99, 108 and 99; mal2  = 92, 101 and 101; mal3 = 91, 87 and 85; mal4 = 75, 90 and 90.

There was a significant effect of the genetic origin (family, *i.e*. female parent) on the masculinization rate in experiment A (S1 Table). It ranged from less than 5% to more than 30% at 8°C and 12°C, and from 7.1% to more than 70% at 18°C (Fig. 3, panels A–D and S1 Table). Whatever the temperature, the rates were similar and low in mal3 and mal4 families and high in mal2 family, whereas mal1 family exhibited intermediate values (S1 Table). In all families, masculinization was increased at 18°C when compared to 8°C or 12°C, though within family (Fig. 3), the increase was significant only for the mal2 progeny (Fig. 3B).

## Discussion

Although the acknowledged system of sex determination in rainbow trout is a genetic sex determination (GSD) system with a male heterogamety (XX-XY) [15], an unexpected masculinization was observed in an all-female rainbow trout population obtained by mitotic gynogenesis [20]. Temperature is known as a major environmental factor able to modulate sex differentiation [7]. Based on preliminary results that suggest the masculinization rate of these XX *mal*-carrying populations was affected by water temperature [Quillet et al., unpublished data], we designed specific experiments to explore the possible effects of temperature on the occurrence of the masculinized phenotypes in *mal*-carrying XX animals. We confirmed in two separate experiments that the rate of masculinization is increased by raising the rearing temperature from low temperatures (8 and 12°C), to a high temperature (18°C). Mortality rates were low during the experiments, and sex differences in mortality cannot explain the differences in masculinization rates we observed. For example, in experiment A, if we hypothesized that all dead fish were males, we would obtain masculinization rates of either 26.4%, 21.5% and 34.5% at 8°C, 12°C and 18°C, respectively. Likewise, if we hypothesized that all dead fish were females, we would obtain masculinization rates of 12.3%, 13.1% and 25.2% at 8°C, 12°C and 18°C, respectively. Therefore, we can conclude that a decrease from 12 to 8°C in the rearing temperature has no significant effect on masculinization while an increase from 12 to 18°C promotes an increase of the masculinization rate of *mal*-carrying rainbow trout. However, because rainbow trout can withstand temperatures lower than 8°C, the effect of even lower temperatures on sex differentiation remains to be explored.

In agreement with previous studies performed with *mal*-carrying animals [20]–[22], we confirmed that the masculinization rates at the reference temperature of 12°C were variable among half-sib progeny (experiment A) and independent progeny (experiment B) depending on the breeding pair, respectively. This result was clearly demonstrated in our common garden experiment, controlled for possible tank effects, in which different progeny exhibited different masculinization rates independent from the temperature effects. Several factors can contribute to differences among progenies. Under the one-locus hypothesis of a recessive *mal* mutation associated to masculinized phenotypes in XX individuals, differences between families in Experiment A are of maternal origin only. However, the actual genotype of the dam cannot be assessed because of the inconsistency of sex reversal of XX*-mal* carrying individuals, as shown in previous study [20]. Moreover, as previously suggested [20], it is likely that more than one locus is involved in the masculinization process of *mal*-carrying XX animals, which can also contribute to genetic differences among families independently of the status at the *mal* locus. Finally, one cannot rule out the hypothesis that non genetic maternal effects, associated to egg components, also contribute to the observed differences. The comparison of sibs from the same dam crossed with different sires would provide further insight to test this hypothesis.

The same kind of family effect on masculinization rates was also observed in response to temperature. Among the different progeny analyzed in our common garden experiment, only the highest temperature (18°C) produced a significant increase in masculinization rate. However, though not significant, an increase in the masculinization rate was also observed in the three other families that similarly had a higher masculinization rate at high temperature and no difference between the 8 and 12°C treatments. It is noteworthy that this significant effect of temperature on masculinization was identified in the progeny with the highest rate of masculinization at 12°C. This was confirmed in experiment B whereby all progeny had a high masculinization rate at 12°C and also exhibited a significant increase of their masculinization rate at 18°C. In salmonids, only two cases of temperature affecting the sex ratio have been reported [12], [13], [16], [19]. In rainbow trout, a high temperature (18°C) treatment similar to the one applied here was shown to modulate sex ratios in both the male and female direction, depending on the population origin and the breeding pair [16]. The existence of a heritable component determining the sex-ratio at high temperature was further demonstrated through one generation of divergent selection producing lines with up- and down-biased sex-ratios [19]. In our experiments, temperature effects have been explored only for masculinization because *mal*-carrying animals are all genetic XX females, but we also observed a family effect on masculinization rates. However, in contrast with previous studies [16], [19], we did not observe any masculinization induced by a high temperature treatment in our all-female control population. Genetic influences on fish TSD have been described in Atlantic silverside [29], and Nile tilapia [3] two species considered prime examples of thermal effects on GSD [7]. Similar genetic influences are likely important in rainbow trout, a species in which some thermal effects on GSD have been already described [16], [19], with in our case, an effect of temperature on GSD combined with the specific *mal* genetic background. This genetic complexity has also been recently suggested for Nile tilapia [4], in which the masculinizing effects of temperature may depend on the combination of different alleles from minor and major sex determining factors. Additional features of the *mal*-carrying line may contribute to its greater susceptibility to temperature than control individuals. Because of the limited number of breeders used to establish the line, genetic drift and increased inbreeding can be suspected to occur in the line. Therefore, the random selection of particular alleles at genes involved in the regulation of temperature effects or reduced homeostasis associated with higher inbreeding could have enhanced the response to temperature during sex differentiation. However, testing this hypothesis would require comparing the effects of temperature in control and *mal*-carrying individuals for other traits than gonad differentiation.

In addition to the various types of masculinization phenotypes and perturbation of gonadal development that were already described in these XX *mal*-carrying populations [20]–[22], we also confirmed the gonadal left-right (LR) asymmetry as previously described [21] with the right gonads being significantly more frequently masculinized than the left gonads at 8 and 12°C. In vertebrates, despite a bilateral symmetrical appearance of the body plan, many internal organs are organized in an asymmetrical LR manner. Gonads are generally regarded as symmetrically paired organs with the important exception of avian species, in which the gonadal LR asymmetry is a well-known phenomenon. This asymmetry can be observed very early during the gonadal development of birds, because the colonization of the gonadal anlage by primordial germ cells (PGCs) is already asymmetrical with a higher number of PGCs found in the left gonad [30]. Apart from birds, a few reports also describe a LR gonadal asymmetry in some mammals [31], [32], amphibians and reptiles (reviewed in [33]). In teleosts, a LR gonadal asymmetry was observed during gonadal differentiation in Argentine silverside, *Odontesthes bonariensis* [34] and also during gonadal development in brown trout, *Salmo trutta* [35]. In some salmonid species, the left gonad also tends to be more developed and less sensitive to steroid treatments [36], [37]. Rainbow trout gonads might then have an underlying asymmetrical differentiation program that is not normally detectable, but that could be revealed in imbalanced physiological conditions, a condition that can occur when a particular genetic background like the *mal*-carrying status induces a delayed or disturbed gonadal differentiation. However, this LR asymmetry is no longer observed in the high temperature treatment that also promotes the highest masculinization rate. This result suggests that temperature not only increases the overall rate of masculinization at the population level but also amplifies each individual masculinization phenotype by shifting slight masculinization phenotypes, including LR intersex, toward a more complete masculinized phenotype.

Thermal effects on GSD in salmonids have been suggested to be the direct consequence of the temperature itself and its magnitude when applied at a critical period of embryonic development [12], [13] instead of the existence of a defined pivotal temperature as described in many reptiles. Such a progressive effect of temperature cannot be totally excluded from our experiments; however this effect would only be detectable above a certain threshold temperature because no significant changes in masculinization rates were detected between 8 and 12°C. Masculinization by high temperatures in fish has been hypothesized to be the result of a physiological stress triggering a cortisol elevation as proposed in TSD fish species including pejerrey, *Odontesthes bonariensis* [38] or in some GSD species with thermal effects including Japanese flounder, *Paralichthys olivaceus* [39] or medaka, *Oryzias latipes* [40]. In addition to these temperature-induced effects, masculinization by direct cortisol treatments was also achieved in rainbow trout [41]. These cortisol treatments induced either a delayed meiotic initiation in Japanese flounder [42] or inhibited the proliferation of germ cells in medaka [40]. Interestingly these two phenotypes were also detected in the gonads of XX *mal*-carrying rainbow trout [22]. However, in rainbow trout, the early stress induced by handling, cold shock [43] or hypoxia [44] did not increase whole body cortisol levels until 6 weeks after hatching, i.e., well after the beginning of the molecular differentiation period of the gonad in this species that begins shortly after hatching [45]. Interestingly, a hypocorticism has been reported [46], [47] in the XX common carp, *Cyprinus carpio*, carrying a masculinization mutation (the *mas* mutation); this is in contrast with the hypothesis that high cortisol levels would trigger masculinization. It should also be noted that this masculinization in XX common carp was always associated with an early meiosis entry in the masculinized animals [48] as opposed to the delayed meiotic initiation in Japanese flounder that is triggered by cortisol levels [42].

In conclusion, our results demonstrate that masculinization in XX *mal*-carrying rainbow trout is increased by high water temperature treatments and also depends on the genetic background of the XX *mal*-carrying fish including at least some clear maternal effect influences. This masculinization in the *mal*-carrying rainbow trout population is potentially triggered by an interaction between the temperature treatment and a complex genetic background likely involving some part of the GSD regulatory cascade along with some minor sex-influencing loci.

## Supporting Information

S1 Figure **Box plots representation of the recorded temperatures for each treatment (8, 12 and 18°C).** The boxes represent the limits of the first and third quartiles (Q1 and Q3) and the red line the median value of the recorded temperatures. Whiskers represent the inferior (I = Q1-1.5(Q3–Q1); black squares) and superior (S = Q3+1.5(Q3–Q1); yellow circles) limits.
(TIF)

S2 Figure **Representative cross-sections of the different gonadal phenotypes.** Normal ovary (A) with no sign of masculinization and normal testis (B). Females with delayed ovarian development (C) scored as “delayed oogenesis females” and intersex gonads (D) showing both testicular and ovarian tissues (D and E). bb  =  Balbiani's body; C =  cysts; EO  =  early meiotic oocytes; fc  =  follicle cell; nu  =  nucleoli; oc  =  ovarian cavity; PO  =  primary growth oocytes; st  =  stroma. Scale bar  = 50 µm.
(TIF)

S1 Table **Frequencies of the gonadal phenotypes in the different families in ExpA.**
(DOCX)
