# Supplementary figures and images for: High Temperature Increases the Masculinization Rate of the All-Female (XX) Rainbow Trout “Mal” Population
Source: PLoS One. 2014 Dec 12;9(12):e113355. doi: 10.1371/journal.pone.0113355 (PMC4264747; doi:10.1371/journal.pone.0113355)

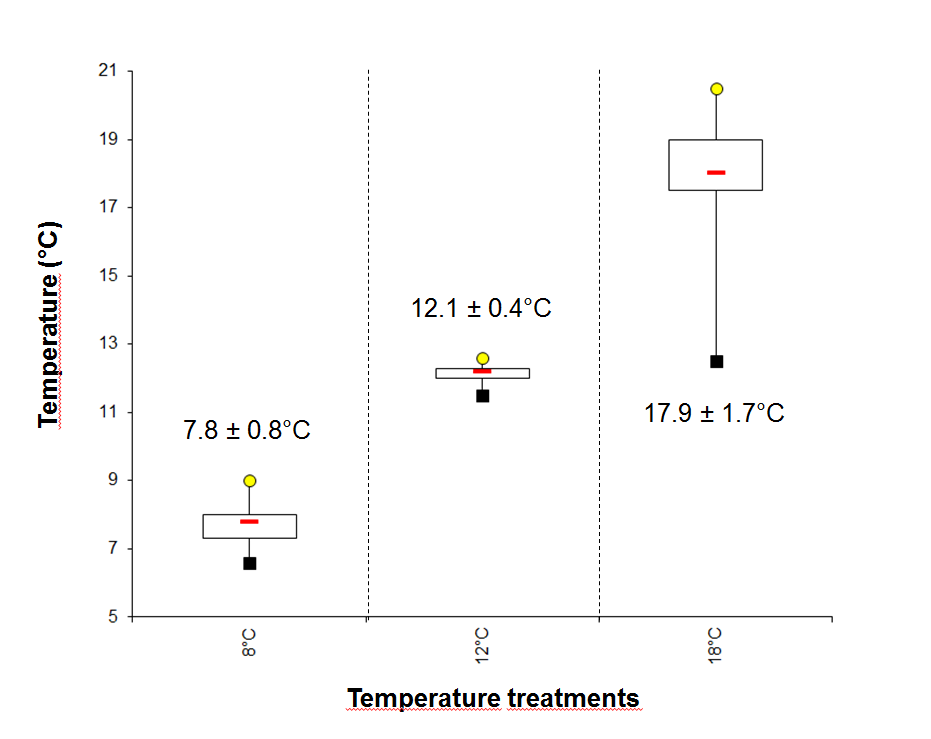

Supplement: S1 Figure — Box plots representation of the recorded temperatures for each treatment (8, 12 and 18°C). The boxes represent the limits of the first and third quartiles (Q1 and Q3) and the red line the median value of the recorded temperatures. Whiskers represent the inferior (I = Q1-1.5(Q3–Q1); black squares) and superior (S = Q3+1.5(Q3–Q1); yellow circles) limits. (TIF) [file pone.0113355.s001.tif]

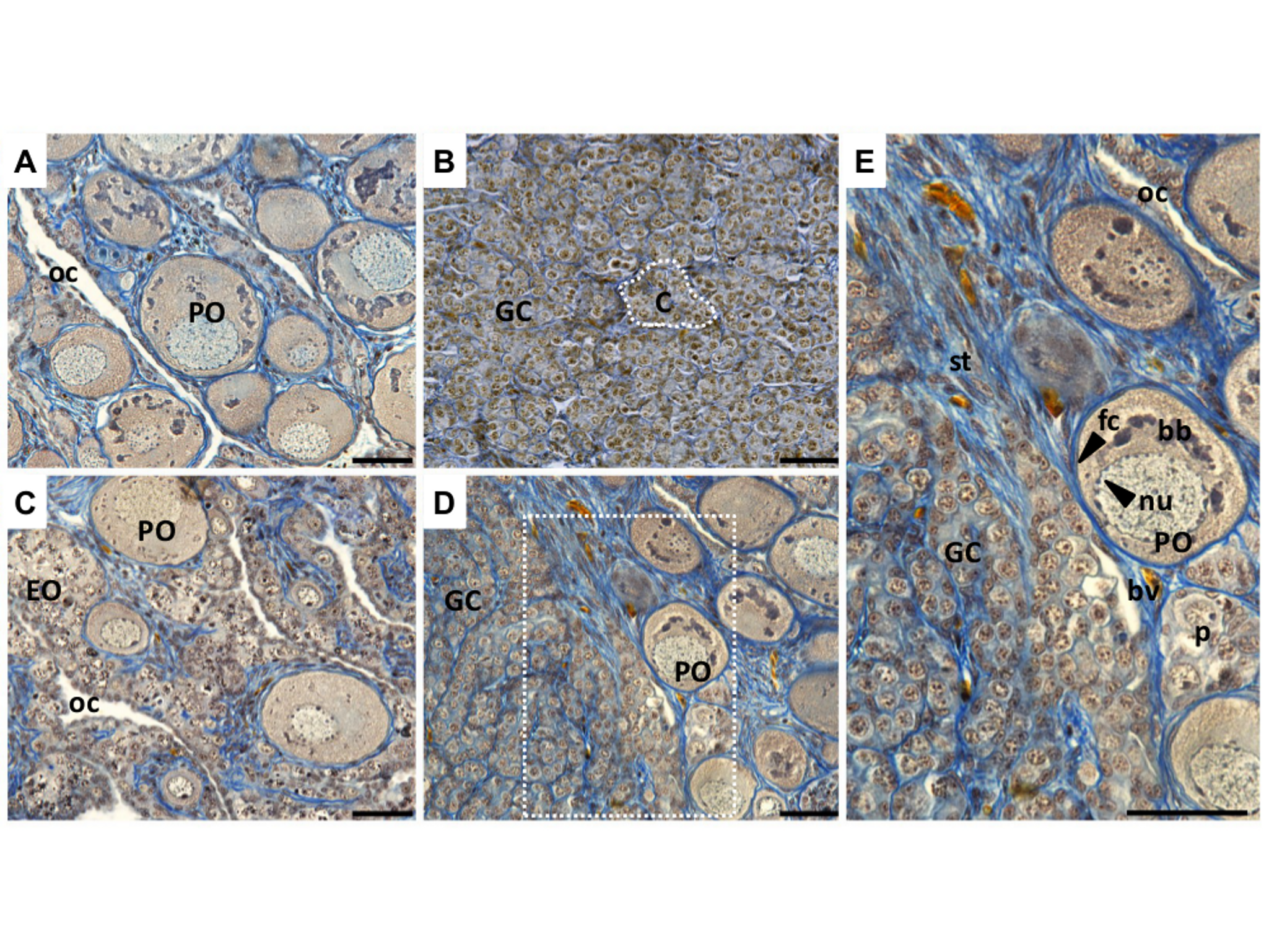

Supplement: S2 Figure — Representative cross-sections of the different gonadal phenotypes. Normal ovary (A) with no sign of masculinization and normal testis (B). Females with delayed ovarian development (C) scored as “delayed oogenesis females” and intersex gonads (D) showing both testicular and ovarian tissues (D and E). bb = Balbiani's body; C = cysts; EO = early meiotic oocytes; fc = follicle cell; nu = nucleoli; oc = ovarian cavity; PO = primary growth oocytes; st = stroma. Scale bar = 50 µm. (TIF) [file pone.0113355.s002.tif]
