# Supplementary material for: High Temperature Increases the Masculinization Rate of the All-Female (XX) Rainbow Trout “Mal” Population
Source: PLoS One. 2014 Dec 12;9(12):e113355. doi: 10.1371/journal.pone.0113355 (PMC4264747; doi:10.1371/journal.pone.0113355)
Supplement: S1 Table — Frequencies of the gonadal phenotypes in the different families in ExpA. (DOCX) [file pone.0113355.s003.docx]

**Table S1: Frequencies of the gonadal phenotypes in the different families in ExpA.**

| Temperature | 8°C | | | | 12°C | | | | 18°C | | | | all | | | | Family effect^1^ |
| --- | --- | --- | --- | --- | --- | --- | --- | --- | --- | --- | --- | --- | --- | --- | --- | --- | --- |
| Family | mal1 | mal2 | mal3 | mal4 | mal1 | mal2 | mal3 | mal4 | mal1 | mal2 | mal3 | mal4 | mal1 | mal2 | mal3 | mal4 |  |
| Number of observations | 99 | 92 | 91 | 75 | 108 | 101 | 87 | 90 | 99 | 101 | 85 | 90 | 306 | 294 | 263 | 255 |  |
| Previtellogenic females (%) | 68.1 | 57.3 | 86.9 | 79.3 | 78.2 | 53.4 | 85.2 | 81.2 | 67.1 | 25.6 | 80.8 | 76.2 | 71.1b | 44.7b | 84.3a | 78.9ab | *P<0.001* |
| Females with delayed oogenesis (%) | 13.2 | 10.7 | 9.1 | 14.1 | 5.7 | 10.0 | 13.0 | 12.9 | 4.7 | 3.3 | 12.1 | 14.9 | 8.0a | 7.8a | 11.5a | 14.0a | *P=0.151* |
| Intersex (%) | 18.7 | 28.0 | 4.0 | 3.3 | 16.1 | 33.3 | 1.8 | 5.9 | 24.7 | 53.3 | 3.0 | 6.9 | 19.8b | 38.9c | 2.9a | 5.4a | *P<0.001* |
| Males(%) | 0 | 4.0 | 0.0 | 3.3 | 0.0 | 3.3 | 0.0 | 0.0 | 3.5 | 17.8 | 4.1 | 2.0 | 1.1a | 8.6b | 1.3b | 1.7b | *P=0.013* |
| Masculinization rate (%) | 18.7 | 32.0 | 4.0 | 6.6 | 16.1 | 36.6 | 1.8 | 5.9 | 28.2 | 71.1 | 7.1 | 8.9 | 20.9c | 47.5c | 4.2a | 7.1b | *P<0.001* |

^1^: test of family effect using Glimmix analysis performed with the whole set of data (3 temperatures and 4 families as fixed effects) on the logit scale assigning binary gonadal phenotypes (1= target gonadal phenotype, 0 = other individuals). Within a line, families with different letters are significantly different for the frequency of the gonadal phenotype (*P<0.05*).
